# Biomarkers predictive of response to pembrolizumab in head and neck cancer

**DOI:** 10.1002/cam4.5434

**Published:** 2022-12-07

**Authors:** David G. Pfister, Robert I. Haddad, Francis P. Worden, Jared Weiss, Ranee Mehra, Laura Q. M. Chow, Stephen V. Liu, Hyunseok Kang, Nabil F. Saba, Lori J. Wirth, Ammar Sukari, Erminia Massarelli, Mark Ayers, Andrew Albright, Andrea L. Webber, Robin Mogg, Jared Lunceford, Lingkang Huang, Razvan Cristescu, Jonathan Cheng, Tanguy Y. Seiwert, Joshua M. Bauml

**Affiliations:** ^1^ Division of Solid Tumor Oncology, Department of Medicine Memorial Sloan Kettering Cancer Center New York New York USA; ^2^ Department of Medical Oncology Dana‐Farber Cancer Institute Boston Massachusetts USA; ^3^ Division of Medical Oncology University of Michigan Ann Arbor Michigan USA; ^4^ Department of Medicine University of North Carolina Lineberger Comprehensive Cancer Center Chapel Hill North Carolina USA; ^5^ Fox Chase Cancer Center Philadelphia Pennsylvania USA; ^6^ University of Maryland Greenebaum Comprehensive Cancer Center Baltimore Maryland USA; ^7^ Department of Medicine, Division of Medical Oncology University of Washington Seattle WA USA; ^8^ The University of Texas at Austin, Dell Medical School Texas Austin USA; ^9^ Department of Medicine Georgetown University Medical Center Washington DC USA; ^10^ Department of Medical Oncology Johns Hopkins University Baltimore Maryland USA; ^11^ University of California San Francisco California USA; ^12^ Department of Hematology and Medical Oncology Winship Cancer Institute, Emory University Atlanta Georgia USA; ^13^ Department of Medicine Massachusetts General Hospital Boston Massachusetts USA; ^14^ Department of Oncology Karmanos Cancer Institute, Wayne State University Detroit Michigan USA; ^15^ Department of Medical Oncology The University of Texas MD Anderson Cancer Center Houston Texas USA; ^16^ Department of Medical Oncology, Merck & Co., Inc. Rahway New Jersey USA; ^17^ Bristol Myers Squibb Philadelphia Pennsylvania USA; ^18^ Section of Hematology‐Oncology University of Chicago Department of Medicine Chicago Illinois USA; ^19^ Johns Hopkins University Baltimore Maryland USA; ^20^ Division of Hematology and Oncology, Department of Internal Medicine University of Pennsylvania Philadelphia Pennsylvania USA; ^21^ Janssen Research and Development Philadelphia Pennsylvania USA

**Keywords:** biomarker, head and neck squamous cell carcinoma, immunotherapy, pembrolizumab, tumor microenvironment, tumor mutational burden

## Abstract

**Background:**

We performed an integrated biomarker evaluation in pembrolizumab‐treated patients with R/M HNSCC enrolled in KEYNOTE‐012 or KEYNOTE‐055. The relationship between biomarkers and HPV status was explored.

**Methods:**

We evaluated PD‐L1 (combined positive score [CPS]), TMB, T‐cell‐inflamed gene expression profile (Tcell_inf_GEP), and HPV status. Associations between biomarkers were evaluated by logistic regression (ORR) and Cox regression (PFS, OS).

**Results:**

Two hundred and fifty‐seven patients (KEYNOTE‐012, *n* = 106; KEYNOTE‐055, *n* = 151) had TMB data available; of these, 254 had PD‐L1 and 236 had Tcell_inf_GEP. TMB, PD‐L1, and Tcell_inf_GEP were each significantly associated with ORR (*p* < 0.01). Kaplan–Meier curves at prespecified cutoffs generally showed PFS and OS separation in the anticipated direction for these biomarkers, except for OS and TMB. TMB did not correlate with PD‐L1 or Tcell_inf_GEP (Spearman *ρ* = −0.03 and *ρ* = −0.13, respectively); PD‐L1 and Tcell_inf_GEP were moderately correlated (Spearman *ρ* = 0.47). In multivariate models, TMB, PD‐L1, and Tcell_inf_GEP were each independently predictive for ORR (*p* < 0.001). ORR was higher in patients with high versus low levels of biomarkers when dichotomized using prespecified cutoffs; patients with higher versus lower levels of TMB and PD‐L1 or TMB and Tcell_inf_GEP had the highest ORRs. Within HPV subgroups, higher versus lower distributions of biomarkers (PD‐L1, TMB, and Tcell_inf_GEP) were associated with response. HPV detection by p16‐immunohistochemistry and WES showed good concordance (81%); results were generally similar by HPV status, regardless of the detection method.

**Conclusions:**

TMB and the inflammatory biomarkers PD‐L1 and Tcell_inf_GEP, assessed alone or together, may be useful for characterizing clinical response to pembrolizumab in R/M HNSCC.

## Graphical abstract

TMB and inflammatory biomarkers PD‐L1 and T‐cell‐inflamed GEP, assessed alone or together, may be useful for characterizing clinical response to pembrolizumab in R/M HNSCC. Greater responses to pembrolizumab were associated with higher levels of TMB and inflammatory biomarkers than were lesser responses, an observation that was consistent regardless of HPV status, suggesting that biomarkers representing complementary measures of tumor antigenicity and a T‐cell‐inflamed tumor microenvironment may be useful in characterizing response to pembrolizumab.

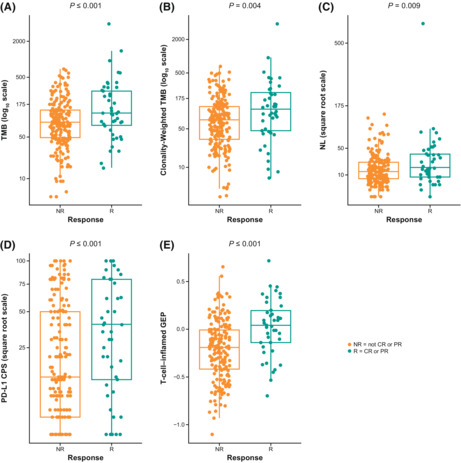

## 1 INTRODUCTION

Immunotherapies that target the programmed death‐1 (PD‐1) axis can provide durable antitumor responses in multiple cancer types; however, the durable benefit is limited to specific patient subpopulations.^1^ Biomarkers indicative of tumor antigenicity, including tumor mutational burden (TMB) and microsatellite instability (MSI), as well as inflammatory biomarkers related to a T‐cell‐inflamed tumor microenvironment, such as programmed death‐ligand 1 (PD‐L1) expression and T‐cell‐activated gene expression signatures, may help characterize patient subpopulations who can benefit from these therapies.^2^ Biomarkers approved by regulatory authorities to predict the likelihood of response to the anti‐PD‐1 monoclonal antibody pembrolizumab include PD‐L1 expression for several tumor types and TMB or MSI regardless of specific cancer.^3^ An 18‐gene T‐cell‐inflamed gene expression profile (Tcell_inf_GEP) can independently predict response to pembrolizumab in multiple tumor types, including head and neck squamous cell carcinoma (HNSCC).^2^, ^4^

Oncogenic viruses such as hepatitis B virus, Merkel cell polyomavirus, Epstein–Barr virus, and human papillomavirus (HPV) generate viral antigens distinct from tumor‐specific neoantigens that arise from somatic mutation and can lead to T‐cell responses.^5^, ^6^ In some virus‐induced cancers, PD‐L1 expression is increased. Higher response rates with immunotherapy have also been noted in virus‐induced versus non‐virus‐induced cancers^6^; however, genetic determinants related to response to immuno‐oncology agents in these tumor types are not well understood. HPV is etiologic for a subset of HNSCC,^7^ and this subset is associated with greater survival than HPV‐negative tumors.^8^ Although HPV status is usually assessed through the detection of p16‐antigen by immunohistochemistry (IHC), HPV genomes can also be detected through genomic methods (i.e., whole‐exome sequencing [WES]).^8^, ^9^

Pembrolizumab demonstrated durable antitumor activity with a manageable safety profile in recurrent and/or metastatic (R/M) HNSCC in the phase Ib KEYNOTE‐012 and phase II KEYNOTE‐055 studies.^10^, ^11^, ^12^ In a recent analysis of KEYNOTE‐012, the expression of TMB, Tcell_inf_GEP, and PD‐L1 each was found to be an independent predictor of clinical response to pembrolizumab.^13^ Patients with high rather than low levels of TMB and inflammatory biomarkers (PD‐L1 expression or Tcell_inf_GEP score) had higher responses to pembrolizumab. The present analysis extends those observations and evaluates the relationships of these biomarkers with response to pembrolizumab and survival using data pooled from KEYNOTE‐012 and KEYNOTE‐055 in patients with R/M HNSCC, including a descriptive evaluation of the relationship between these biomarkers and HPV status.

## 2 METHODS

### 2.1 Study design and patients

The study design and eligibility criteria of the multicohort phase Ib KEYNOTE‐012 (ClinicalTrials.gov, NCT01848834) study and the phase II KEYNOTE‐055 (ClinicalTrials.gov, NCT02255097) study have been reported.^10^, ^11^, ^12^ In brief, eligible patients in KEYNOTE‐012 cohorts B and B2 had confirmed R/M HNSCC by Response Evaluation Criteria in Solid Tumors, version 1.1, by investigator review and Eastern Cooperative Oncology Group performance status (ECOG PS) ≤1; cohort B enrolled 60 patients with PD‐L1‐positive tumors (≥1%, QualTek IHC), and 132 patients with PD‐L1‐unselected tumors were enrolled in cohort B2. Eligible patients in KEYNOTE‐055 had confirmed R/M HNSCC of the oral cavity, oropharynx, hypopharynx, or larynx resistant to platinum and to cetuximab; had an ECOG PS of 0 or 1; and provided newly obtained core or excisional biopsy for PD‐L1 expression analysis (*n* = 171). Concurrent treatment with platinum and cetuximab was not required; however, patients must have experienced progressive disease (PD) or recurrence <6 months after the last dose of each therapy. For both studies, key exclusion criteria included previous treatment with an anticancer monoclonal antibody <4 weeks of the initiation of study drug; previous chemotherapy, small molecule‐targeted therapy, or radiation therapy <2 weeks of the initiation of study drug; known active central nervous system metastases; and a diagnosis of immunodeficiency, autoimmune disease, interstitial lung disease, or active infection that required systemic therapy.

In KEYNOTE‐012, patients received pembrolizumab 10 mg/kg every 2 weeks in cohort B and 200 mg every 3 weeks in cohort B2. In KEYNOTE‐055, patients received pembrolizumab 200 mg every 3 weeks. In both studies, treatment continued for ≤2 years or until confirmed PD or unacceptable toxicity, investigator decision, or withdrawal of patient consent.

The study protocols were approved by regulatory boards or ethics review committees at each study center. The studies were conducted in accordance with the Declaration of Helsinki and Good Clinical Practice guidelines. All patients provided written informed consent before study entry.

### 2.2 Assessments

TMB was assessed using WES as previously described.^2^ PD‐L1 expression level was assessed via IHC using PD‐L1 IHC 22C3 pharmDx (Agilent) and measured by combined positive score (CPS), defined as the number of PD‐L1‐positive cells (tumor cells, lymphocytes, macrophages) divided by the total number of viable tumor cells, multiplied by 100.^14^ For the Tcell_inf_GEP, RNA was assessed using the NanoString platform and calculated as a weighted sum of normalized expression values for the 18 genes (*TIGIT*, *CD27, CD8A, PDCD1LG2* [PD‐L2], *LAG3*, *CD274* [PD‐L1], *CXCR6*, *CMKLR1*, *NKG7*, *CCL5*, *PSMB10*, *IDO1*, *CXCL9, HLA.DQA1*, *CD276*, *STAT1*, *HLA.DRB1*, *HLA.E*).^4^ HPV status was determined by p16‐IHC for patients with cancer of the oropharynx. p16‐IHC testing was not conducted for primary tumor locations outside the oropharynx, and such tumors were categorized as HPV negative. HPV status was also assessed by WES regardless of the tumor site.

### 2.3 Statistical analysis

Statistical testing of biomarker and response relationships, prespecified in the statistical analysis plans for KEYNOTE‐012 and KEYNOTE‐055, was performed using data pooled from both studies. TMB, PD‐L1 expression, and Tcell_inf_GEP score relationships with objective response rate (ORR; defined as the proportion of patients achieving complete response or partial response), progression‐free survival (PFS), and overall survival (OS) with pembrolizumab were assessed in all patients and by HPV status. Associations between TMB, PD‐L1, Tcell_inf_GEP, neoantigen load, and clonality‐weighted TMB (TMB × clonality as calculated using a published algorithm^15^) and ORR were assessed by logistic regression. Associations of TMB, PD‐L1, and Tcell_inf_GEP with PFS and OS were assessed by Cox regression. Associations of neoantigen load and tumor clonality (an estimate of TMB restricted to clonal mutations) with ORR were also evaluated. An appropriate transformation for TMB, PD‐L1, neoantigen load, and clonality‐weighted TMB, either log or square root, was applied when needed. Models were adjusted for ECOG PS and study or cohorts. One‐sided nominal *p* values were reported for ORR, PFS, and OS because a positive association was hypothesized. Significance was determined at the 0.05 level, unadjusted for multiplicity. The area under the receiver‐operating characteristic (AUROC) curve was used as a measure of the discriminatory ability of TMB, PD‐L1, and Tcell_inf_GEP biomarkers to distinguish responders from nonresponders. The following cutoffs were used to assess the clinical utilities of the biomarkers with the response (1) 175 mutations/exome (mut/exome) for TMB, derived from TMB and GEP data across multiple cohorts^16^ (previously shown to be concordant with 10 mut/Mb [FoundationOne®CDx])^17^; (2) CPS 1 for PD‐L1 expression^14^; and (3) –0.318 for GEP score, defined using multiple tumor types^18^ and preceding the availability of gene expression data from KEYNOTE‐055.

Correlations between TMB and inflammatory biomarkers (PD‐L1 and Tcell_inf_GEP) were assessed using Spearman correlation. A contingency table was used to evaluate the concordance of HPV status (WES versus p16 IHC). Boxplots were used to descriptively illustrate the distribution of each biomarker (TMB/Tcell_inf_GEP/PD‐L1) by HPV status; the mean difference of each biomarker in HPV‐positive versus HPV‐negative subgroups was tested using a two‐sample *t* test, and the adjusted *p* values are reported for multiple testing across the three biomarkers (TMB/Tcell_inf_GEP/PD‐L1). The Hochberg step‐up procedure was used for multiplicity to control the family‐wise error rate. Testing for differential biomarker relationships according to HPV status was performed with an interaction term between the biomarker (TMB/Tcell_inf_GEP/PD‐L1) and HPV status in a logistic regression model (other terms were ECOG PS, study/cohort, the biomarker itself [TMB/Tcell_inf_GEP/PD‐L1], and HPV status) and was similarly adjusted for multiplicity.

## 3 RESULTS

### 3.1 Patients

Of 363 total patients from the KEYNOTE‐012 and KEYNOTE‐055 trials, 257 patients had evaluable TMB data (106, KEYNOTE‐012; 151, KEYNOTE‐055); of these, 254 had available PD‐L1 data and 236 had available Tcell_inf_GEP data. The baseline characteristics of the patients with available TMB data were generally similar to those of the total population (Table 1). Median follow‐up in KEYNOTE‐012 was 14 months (interquartile range, 4–14) in cohort B and 9 months (interquartile range, 3–11) in cohort B2, and median follow‐up in KEYNOTE‐055 was 7 months (range, 0–17).^10^, ^11^, ^12^

**TABLE 1:** Baseline characteristics of biomarker population

Characteristic	Overall study population^a^ *N* = 363	WES population^b^ *n* = 257
Age, years, median (range)	61 (20 to 90)	61 (25 to 90)
Male, *n* (%)	297 (82)	207 (81)
ECOG PS (1 or 2), *n* (%)	258 (71)	178 (69)
Stage M1 disease, *n* (%)	321 (88)	230 (89)
No. of previous lines of therapy, *n* (%)		
0	37 (10)	24 (9)
1	80 (22)	49 (19)
2	113 (31)	93 (36)
≥3	133 (37)	91 (35)
HPV positive by p16‐IHC, *n* (%)	82 (23)	57 (22)
HPV positive by WES, *n* (%)	—	79 (31)

^a^ Overall study cohort was composed of 192 (KEYNOTE‐012) and 171 patients (KEYNOTE‐055).

^b^ Patients with available WES data included 106 (KEYNOTE‐012) and 151 patients (KEYNOTE‐055).

### 3.2 Association of biomarkers with ORR

**FIGURE 1:**
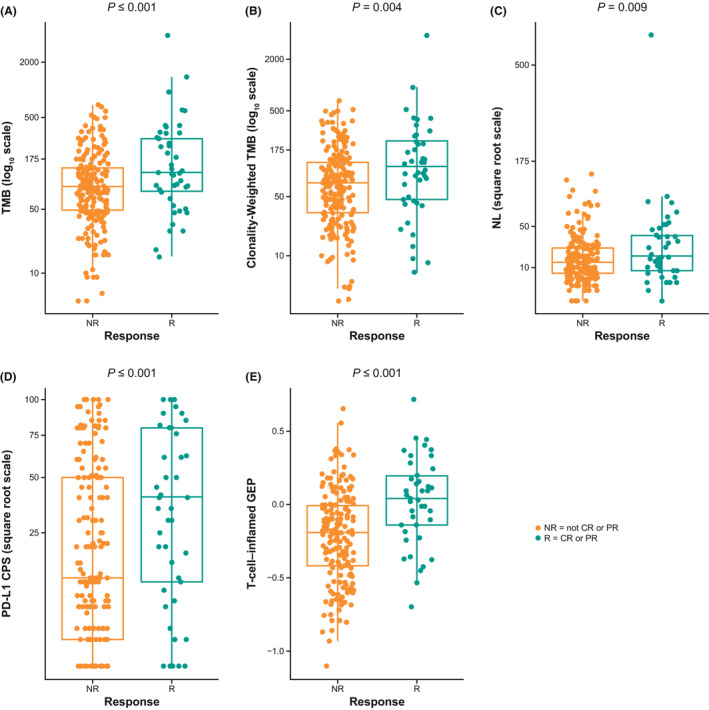
Association between biomarkers and response in all patients. (A) TMB, (B) TMB weighted by clonality, (C) neoantigen load, (D) PD‐L1 CPS, and (E) Tcell_inf_GEP

**FIGURE 2:**
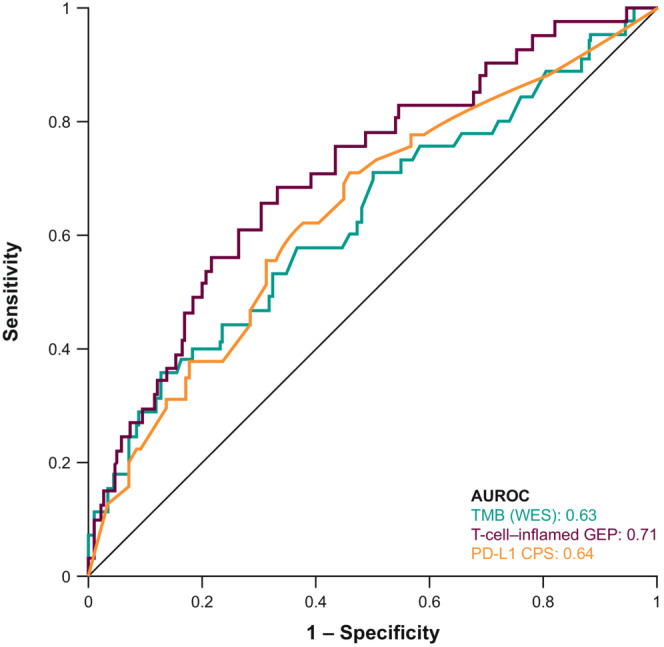
AUROC curve of TMB, PD‐L1 CPS, and Tcell_inf_GEP in all patients

TMB, PD‐L1, and Tcell_inf_GEP were each significantly associated with ORR (*p* ≤ 0.001) (Figure 1). Clonality‐weighted TMB and neoantigen load were highly correlated with TMB (Spearman *ρ* = 0.90 and *ρ* = 0.85), and increasing neoantigen load and clonality‐weighted TMB were positively associated with ORR in all patients (*p* = 0.009 and *p* = 0.004) (Figure 1). Predictive discriminatory utility scores for ORR of TMB, PD‐L1, and Tcell_inf_GEP by AUROC analysis were similar: 0.63 (95% CI, 0.53–0.73), 0.64 (95% CI, 0.55–0.73), and 0.71 (95% CI, 0.62–0.80), respectively (Figure 2); the AUROC was 0.62 (95% CI, 0.52–0.72) for clonality‐weighted TMB. Of importance, the Tcell_inf_GEP score was initially identified with a multitumor, 220‐patient training data set that included 40 patients from KEYNOTE‐012 cohort B1 and that later was independently validated using data from KEYNOTE‐012 cohort B2^4^; thus, some upward bias in the Tcell_inf_GEP AUROC estimate might have occurred because of the inclusion of training data.

### 3.3 Joint assessment of biomarkers

**FIGURE 3:**
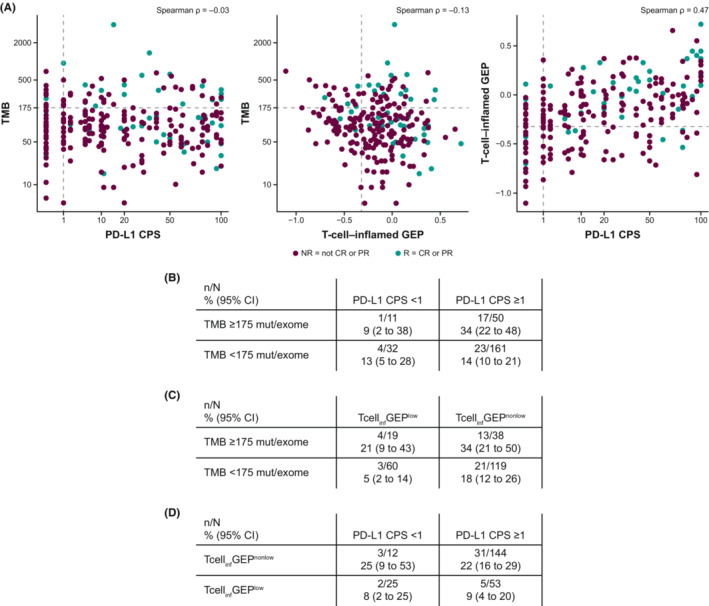
Correlation between (A) PD‐L1 CPS and TMB, or Tcell_inf_GEP and TMB, or PD‐L1 CPS and Tcell_inf_GEP and the response rate (95% CI) of the dual biomarkers (B) TMB and PD‐L1 CPS, (C) TMB and Tcell_inf_GEP, and (D) Tcell_inf_GEP and PD‐L1 CPS in all patients. Data in panels B, C, and D are shown for patients who had data available for both biomarkers

No notable correlation was observed between TMB and inflammatory biomarkers (Spearman ρ = −0.03 for PD‐L1 and ρ = −0.13 for Tcell_inf_GEP) (Figure 3A), whereas PD‐L1 and Tcell_inf_GEP were moderately correlated (Spearman *ρ* = 0.47) (Figure 3A). When TMB was assessed in a multivariate model with either PD‐L1 or Tcell_inf_GEP, TMB and each marker of inflammation remained significantly predictive (all *p* ≤ 0.001) (Table S1).

Clinical response was also evaluated based on prespecified cutoffs for each biomarker. The response was higher in the TMB ≥175 mut/exome subgroup than in the TMB <175 mut/exome subgroup (18/61 patients [30%] vs. 27/196 patients [14%]), in the PD‐L1 CPS ≥1 subgroup than in the PD‐L1 CPS <1 subgroup (40/211 patients [19%] vs. 5/43 patients [12%]), and in the Tcell_inf_GEP^nonlow^ (≥−0.318) subgroup than in the Tcell_inf_GEP^low^ (<−0.318) subgroup (34/157 patients [22%] vs. 7/79 patients [9%]). The response was highest in the subgroup of patients with both TMB ≥175 mut/exome and PD‐L1 CPS ≥1 (17/50 patients [34%]) (Figure 3B) and in the subgroup of patients with TMB ≥175 mut/exome and Tcell_inf_GEP^nonlow^ (13/38 patients [34%]) (Figure 3C).

### 3.4 Association of biomarkers with PFS and OS

**FIGURE 4:**
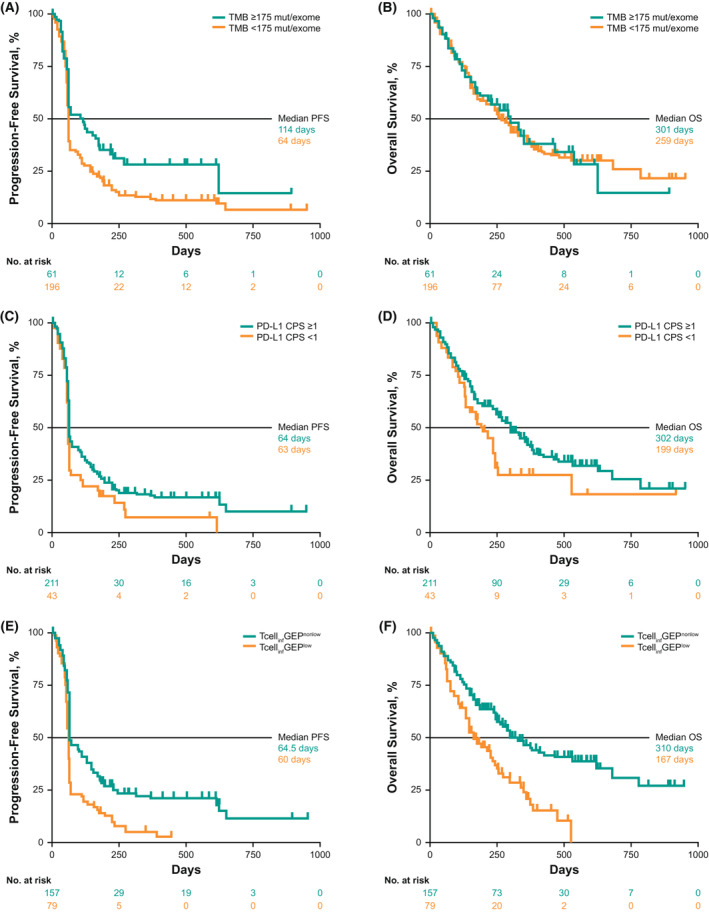
Association between biomarkers and PFS and OS in all patients at prespecified cutoffs. (A) TMB and PFS, (B) TMB and OS, (C) PD‐L1 CPS and PFS, (D) PD‐L1 CPS and OS, (E) Tcell_inf_GEP and PFS, and (F) Tcell_inf_GEP and OS

Median PFS was 114 days (95% CI, 63–180) in the TMB ≥175 mut/exome subgroup versus 64 days (95% CI, 63–64) in the TMB <175 mut/exome subgroup, 64 days (95% CI, 63–71) in the PD‐L1 CPS ≥1 subgroup versus 63 days (95% CI, 61–66) in the PD‐L1 CPS <1 subgroup, and 64.5 days (95% CI, 64–111) in the Tcell_inf_GEP^nonlow^ subgroup versus 60 days (95% CI, 58–64) in the Tcell_inf_GEP^low^ subgroup (Figure 4).

Median OS was 301 days (95% CI, 178 to not evaluable) in the TMB ≥175 mut/exome subgroup versus 259 days (95% CI, 240–354) in the TMB <175 mut/exome subgroup, 302 days (95% CI, 256–365) in the PD‐L1 CPS ≥1 subgroup versus 199 days (95% CI, 134–255) in the PD‐L1 CPS <1 subgroup, and 310 days (95% CI, 259–540) in the Tcell_inf_GEP^nonlow^ subgroup versus 167 days (95% CI, 136–246) in the Tcell_inf_GEP^low^ subgroup (Figure 4).

### 3.5 Distribution of biomarkers and by HPV status

Of 363 patients, 256 (71%) had evaluable WES and p16‐IHC data. Despite p16‐IHC analysis being restricted to oropharyngeal tumors and WES analysis performed on all tumor sites, detection of HPV status by p16‐IHC and WES showed good concordance (208/256 [81%]) (Table S2) in the analysis population of patients with evaluable WES data. Similar proportions of patient tumors were classified as HPV negative (78% and 69%) and HPV positive (22% and 31%) by p16‐IHC and WES, respectively.

The distribution of biomarkers (TMB/PD‐L1/Tcell_inf_GEP) was comparable in HPV‐positive and HPV‐negative subgroups, defined by either WES or p16‐IHC (Figure S1A–C). Two‐sample *t* testing showed no significant difference between HPV‐positive and HPV‐negative subgroups, defined by either WES or p16‐IHC for any of the biomarkers (adjusted *p >* 0.3).

### 3.6 Association of biomarkers with ORR by HPV status

Evaluating trends for TMB, PD‐L1, and Tcell_inf_GEP within HPV status suggested consistency in the positive association between each biomarker and response in HPV‐positive and HPV‐negative subgroups (Figures S2A–C). Some associations with outcome appeared to be stronger in one HPV subgroup than another. For example, TMB distribution was more separated for responders than nonresponders in the HPV‐negative group and the HPV‐positive group. Conversely, PD‐L1 CPS and Tcell_inf_GEP distributions showed stronger trends with response status for the HPV‐positive group than the HPV‐negative group. Statistical interaction testing was conducted for HPV‐specific associations for these three biomarkers with ORR, albeit in a manner outside the statistical analysis plan of each study, to gauge the evidence supporting differences in the relationship between TMB and inflammatory biomarkers with clinical outcome according to HPV status based on a more powered analysis offered by the combined two‐study data package. The results of this interaction testing, with multiplicity adjustment for the three biomarkers within HPV assay type, provided some indication that the association between the inflammatory markers PD‐L1 and Tcell_inf_GEP might have been stronger in the HPV‐positive subgroup, although the two versions of the HPV assays were not consistent in their testing conclusions with regard to adjusted *P* values achieving significance at the 0.05 level (Table S3).

## 4 DISCUSSION

In this exploratory analysis of patients with R/M HNSCC enrolled in KEYNOTE‐012 and KEYNOTE‐055 who had evaluable WES data, TMB and the inflammatory biomarkers PD‐L1 CPS and Tcell_inf_GEP were independently predictive of ORR to pembrolizumab. Patients with high levels (based on a prespecified cutoff) of TMB, PD‐L1, and Tcell_inf_GEP versus low levels of these biomarkers had higher response rates to pembrolizumab, and patients whose tumors expressed high levels of dual biomarkers (both TMB and PD‐L1 CPS and TMB and Tcell_inf_GEP) had the best responses to pembrolizumab. Taken together, the data suggest that these biomarkers reflect complementary measures of tumor antigenicity and an inflamed tumor microenvironment and that, used alone or jointly, have the potential to characterize responses to anti‐PD‐1 therapy in HNSCC. However, outlier responses, such as patients whose tumors express low levels of TMB and PD‐L1, suggest that additional biology or dynamic changes may also contribute to these effects, necessitating further evaluation in additional studies.

The results of this analysis are consistent with those of previous studies showing that TMB, PD‐L1, and Tcell_inf_GEP are related to response to anti‐PD‐1 therapy in multiple cancers^2^ and with those of a recent analysis of the KEYNOTE‐012 trial in patients with HNSCC.^13^ PD‐L1 is a US Food and Drug Administration‐approved diagnostic biomarker that is related to response to pembrolizumab monotherapy in several cancers, including HNSCC when used as first‐line therapy,^3^ and is considered to be driven by interferon‐gamma signaling as partly indicative of a T‐cell‐inflamed tumor microenvironment. In a similar fashion, Tcell_inf_GEP, a signature composed of genes related to multiple cell types involved in the cytolytic process, including PD‐L1 and PD‐L2 as member genes whose levels are coexpressed with those of other genes in the signature, is related to response to pembrolizumab.^4^ Hence, as expected and as demonstrated previously,^19^ PD‐L1 and Tcell_inf_GEP were moderately positively correlated in this analysis. Similar to other published data,^2^, ^13^ this combined HNSCC cohort shows TMB did not correlate with PD‐L1 or Tcell_inf_GEP but rather indicates mutational load acted as an additional explanatory axis for objective response to pembrolizumab in this pooled analysis. Pembrolizumab was recently approved by the US Food and Drug Administration to treat patients with unresectable or metastatic TMB‐high (≥10 mut/Mb) solid tumors who experienced PD after previous treatment.^3^, ^20^

Using prespecified biomarker cutoffs, trends of longer median PFS were observed with higher versus lower levels of TMB but not PD‐L1 or Tcell_inf_GEP. Evaluation of the Kaplan–Meier curves shows a fairly similar level of progression early, regardless of biomarker status, but later separation in PFS curves in the anticipated direction according to the biomarker cutoff (Figure 4). Longer median OS was associated with higher rather than lower levels of PD‐L1 and Tcell_inf_GEP but not of TMB. However, OS data from single‐arm studies should be interpreted with caution; a more informative evaluation of the relationship between OS and TMB will require larger randomized studies.

HPV infection is a risk factor for some HNSCC subtypes,^21^ and its presence can be determined by p16‐IHC, DNA/RNA‐in situ hybridization, or genomic methods (e.g., WES). In our study, both p16‐IHC and WES showed good concordance (81%) in HPV detection, and results in the HPV subgroups identified by either method were generally similar. When evaluating the response to pembrolizumab by HPV status in patients with HNSCC in KEYNOTE‐012 and KEYNOTE‐055, both studies previously reported that response rates were similar regardless of HPV status.^10^, ^11^, ^12^ In the current analysis, the distributions of TMB, PD‐L1, and Tcell_inf_GEP were similar among the HPV‐positive and HPV‐negative subgroups. The inflammatory biomarkers PD‐L1 and Tcell_inf_GEP, TMB, neoantigen load, and clonality‐weighted TMB were each associated with response to pembrolizumab regardless of HPV status detected by p16 or WES. Higher response rates were observed in patients with high levels of TMB or either of the inflammatory biomarkers in both HPV subgroups. It is possible that biomarker trends with the clinical outcome may vary by HPV status. For the trends observed here, statistical significance and clinical relevance were not clear but may be worth additional follow‐up as further data accumulate.

In conclusion, TMB and the inflammatory biomarkers PD‐L1 CPS and Tcell_inf_GEP were each significantly and independently predictive of response to pembrolizumab in patients with HNSCC. Greater responses to pembrolizumab were associated with higher levels of the inflammatory biomarkers and TMB than were lesser responses, an observation that was consistent regardless of HPV status, suggesting that biomarkers representing complementary measures of tumor antigenicity and a T‐cell‐inflamed tumor microenvironment may be useful in characterizing clinical response to pembrolizumab in HNSCC. Larger randomized studies are required to better identify and understand biomarkers of response and resistance to pembrolizumab monotherapy and combination therapy in patients with HNSCC.

## AUTHOR CONTRIBUTIONS

**David G. Pfister:** Validation (equal); writing – review and editing (equal). **Robert I. Haddad:** Data curation (equal); formal analysis (equal); validation (equal); writing – review and editing (equal). **Francis P. Worden:** Validation (equal); writing – review and editing (equal). **Jared Weiss:** Conceptualization (equal); data curation (equal); investigation (equal); validation (equal); writing – review and editing (equal). **Ranee Mehra:** Data curation (equal); validation (equal); writing – review and editing (equal). **Laura Q. M. Chow:** Data curation (equal); formal analysis (equal); validation (equal); writing – original draft (equal); writing – review and editing (equal). **Stephen V. Liu:** Data curation (equal); formal analysis (equal); validation (equal); writing – review and editing (equal). **Hyunseok Kang:** Data curation (equal); validation (equal); writing – review and editing (equal). **Nabil F. Saba:** Data curation (equal); validation (equal); writing – review and editing (equal). **Lori J. Wirth:** Data curation (equal); formal analysis (equal); validation (equal); writing – review and editing (equal). **Ammar Sukari:** Formal analysis (equal); validation (equal); writing – review and editing (equal). **Erminia Massarelli:** Data curation (equal); validation (equal); writing – review and editing (equal). **Mark Ayers:** Conceptualization (equal); investigation (equal); writing – original draft (equal); writing – review and editing (equal). **Andrew Albright:** Conceptualization (equal); data curation (equal); investigation (equal); writing – review and editing (equal). **Andrea L. Webber:** Validation (equal); writing – review and editing (equal). **Robin Mogg:** Formal analysis (equal); writing – review and editing (equal). **Jared Lunceford:** Formal analysis (equal); validation (equal); writing – original draft (equal); writing – review and editing (equal). **Lingkang Huang:** Conceptualization (equal); formal analysis (equal); investigation (equal); validation (equal); writing – original draft (equal); writing – review and editing (equal). **Razvan Cristescu:** Conceptualization (equal); formal analysis (equal); investigation (equal); validation (equal); writing – original draft (equal); writing – review and editing (equal). **Jonathan Cheng:** Conceptualization (equal); data curation (equal); investigation (equal); writing – review and editing (equal). **Tanguy Y. Seiwert:** Data curation (equal); validation (equal); writing – review and editing (equal). **Joshua M. Bauml:** Conceptualization (equal); data curation (equal); formal analysis (equal); investigation (equal); validation (equal); writing – original draft (equal); writing – review and editing (equal).

## FUNDING INFORMATION

These studies were supported by Merck Sharp & Dohme LLC, a subsidiary of Merck & Co., Inc., Rahway, NJ, USA. The funder collaborated jointly with the academic authors to design the study and gather, analyze, and interpret the results. All authors had full access to all study data and had final responsibility for the decision to submit the manuscript for publication. The sponsor funded medical writing and/or editorial assistance for this manuscript.

## CONFLICT OF INTEREST

DGP reports grants (clinical trial support) from AZ/MedImmune and Hookipa, personal fees (Data and Safety Monitoring Board member) from Boehringer Ingelheim, and personal fees (services other than consulting) from Incyte. RIH reports employment at Dana‐Farber Cancer Institute; leadership for the National Comprehensive Cancer Network; a consulting or advisory role for Celgene, Merck, Eisai, Bristol Myers Squibb, AstraZeneca, Pfizer, Loxo, Genentech, Immunomic Therapeutics, GlaxoSmithKline, Gilead Sciences, Vaccinex, EMD Serono, BioNTech AG, Achilles Therapeutics, Bayer, and Mirati Therapeutics; researching funding (to institution) from Boehringer Ingelheim, Merck, Bristol Myers Squibb, Celgene, AstraZeneca, VentiRx, Genentech, Pfizer, and Kura; other relationships with Nanobiotix and ISA Pharmaceuticals. FPW reports personal fees (advisory board and clinical trial support) from Merck; grants and personal fees (advisory board and clinical trial support) from Exelixis, Eli Lilly, and Eisai; grants (clinical trial support) from Pfizer; and personal fees (advisory board) from Bristol Meyers Squibb. JW has nothing to disclose. RM reports personal fees (advisory board) from Rakuten Medical and others (researching funding) from Merck and AstraZeneca. LQMC reports grants and personal fees from Merck (de minimus personal honoraria for lung cancer immunotherapy advisory board over the last 5 years, not directly associated with current topic; prior institution [University of Washington from 2012 to 2019] received grant funding for study enrollment and conduct); grants (research funding to prior institution) from Lilly/ImClone, Bristol Myers Squibb, AstraZeneca/MedImmune, Pfizer, Seattle Genetics, Dynavax, Alkermes, and Novartis; grants (current institutional study research funding) from Alkermes; personal fees (advisory board, lung cancer [spring 2021]) from AstraZeneca/MedImmune; personal fees (advisory board, cancer cachexia [2019]) from Pfizer; personal fees (advisory board, immunotherapy [2019]) from Dynavax; personal fees (advisory board, immunotherapy [2020]) from Alkermes; personal fees (brief consultation – de minimus honoraria 2019, 2020) from Cullinan; personal fees (brief consultation – de minimus honoraria 2020) from Elicio; personal fees (research funding to prior institution) from Genentech; personal fees (de minimus advisory board honoraria and study chair [2013–2019]; advisory board, targeted therapy lung cancer [2020]) from Novartis; personal fees (advisory board and consulting, lung cancer targeted therapy – de minimus payment 2020) from Daiichi Sankyo; personal fees (virtual advisory board, head and neck cancer and CD47 therapy di minimus payment November 2020) from Gilead; personal fees (virtual advisory board, immunotherapy and cutaneous skin cancer; advisory board di minimus payment December 2020) from Regeneron; personal fees (virtual advisory board, small cell lung cancer [spring 2021]) from Ipsen; personal fees (virtual targeted therapy lung cancer advisory board [spring 2021]) from Blueprint; personal fees (virtual advisory board [June 2021]) from Nanobiotix; personal fees (di minimus virtual advisory board, lung cancer [June 2021]) from Sanofi‐Genzyme; and personal fees (de minimus virtual lung cancer advisory board [June 2021]) from BeiGene. SVL reports grants from Alkermes, AstraZeneca, Bayer, Blueprint, Bristol Myers Squibb, Elevation Oncology, Genentech, Lilly, Merck, Merus, Pfizer, Rain Therapeutics, RAPT, Takeda, and Turning Point Therapeutics; and personal fees from Amgen, AstraZeneca, BeiGene, Blueprint, Bristol Myers Squibb, Daiichi Sankyo, Eisai, Elevation Oncology, Genentech, Guardant Health, Inivata, Janssen, Jazz Pharmaceuticals, Eli Lilly, Merck, Novartis, Pfizer, Regeneron, Takeda, and Turning Point Therapeutics. HK reports personal fees (consulting fee) from Pin Therapeutics; other (data safety monitoring committee) from MitoImmune; and other (advisory board) from MitoImmune, Bayer, Exelixis, and Achilles Therapeutics. NFS reports personal fees (advisory role) from Merck, GlaxoSmithKline, and Kura and grants (funding for research) from BMS and Exelixis. LJW reports personal fees (advisory board) from Merck, Bayer HealthCare, Blueprint Medicines, Eli Lilly, Exelixis, Genentech USA, and Loxo Oncology; and personal fees (sits on Data and Safety Monitoring Committee) from Iovance Biotherapeutics and PSD Biotechnology. AS reports personal fees (speaker fees) from Merck. EM reports personal fees (speakers bureau) from AstraZeneca, Eli Lilly, Merck, and Takeda and personal fees (advisory board) from BMS, Genentech, Eli Lilly, Janssen, Merck, Mirati, and Sanofi. MA is an employee of Merck Sharp & Dohme LLC, a subsidiary of Merck & Co., Inc., Rahway, NJ, USA, and a stockholder of Merck & Co., Inc., Rahway, NJ, USA. AA is an employee of Merck Sharp & Dohme LLC, a subsidiary of Merck & Co., Inc., Rahway, NJ, USA. ALW is an employee of Merck Sharp & Dohme LLC, a subsidiary of Merck & Co., Inc., Rahway, NJ, USA. RM is an employee of Merck Sharp & Dohme LLC, a subsidiary of Merck & Co., Inc., Rahway, NJ, USA. JL is an employee of Merck Sharp & Dohme LLC, a subsidiary of Merck & Co., Inc., Rahway, NJ, USA, and a stockholder of Merck & Co., Inc., Rahway, NJ, USA. LH is an employee of Merck Sharp & Dohme LLC, a subsidiary of Merck & Co., Inc., Rahway, NJ, USA, and a stockholder of Merck & Co., Inc., Rahway, NJ, USA. RC is an employee of Merck Sharp & Dohme LLC, a subsidiary of Merck & Co., Inc., Rahway, NJ, USA, and a stockholder of Merck & Co., Inc., Rahway, NJ, USA. JC was an employee of Merck Sharp & Dohme LLC, a subsidiary of Merck & Co., Inc., Rahway, NJ, USA at the time of this study. TYS reports grants (other clinical trials) from Merck/MSD, BMS, Genentech/Roche, Regeneron, AstraZeneca, Cue Biopharma, Nektar, and KURA; other (education, advisory board) from Merck/MSD; other (advisory board) from Regeneron, Cue Biopharma, KURA, and Innate; and other (steering committee) from AstraZeneca and Nektar. JMB reports grants from Merck, Clovis, Carevive Systems, Novartis, Bayer, Janssen, AstraZeneca, Takeda, and Carisma Therapeutics; and personal fees from Clovis, Bristol Meyers Squibb, Astra Zeneca, Celgene, Boehringer Ingelheim, Janssen, Merck, Guardant Health, Genentech, Takeda, Ayala, Regeneron, Inivata, and Novartis.

## ETHICAL APPROVAL STATEMENT

The study protocols were approved by regulatory boards or ethics review committees at each study center. The studies were conducted in accordance with the Declaration of Helsinki and Good Clinical Practice guidelines. All patients provided written informed consent before study entry.

## Supporting information


Appendix S1

## Data Availability

Merck Sharp & Dohme LLC, a subsidiary of Merck & Co., Inc., Rahway, NJ, USA (MSD) is committed to providing qualified scientific researchers access to anonymized data and clinical study reports from the company's clinical trials for the purpose of conducting legitimate scientific research. MSD is also obligated to protect the rights and privacy of trial participants and, as such, has a procedure in place for evaluating and fulfilling requests for sharing company clinical trial data with qualified external scientific researchers. The MSD data‐sharing website (available at: http://engagezone.msd.com/ds_documentation.php) outlines the process and requirements for submitting a data request. Applications will be promptly assessed for completeness and policy compliance. Feasible requests will be reviewed by a committee of MSD subject matter experts to assess the scientific validity of the request and the qualifications of the requestors. In line with data privacy legislation, submitters of approved requests must enter into a standard data‐sharing agreement with MSD before data access is granted. Data will be made available for request after product approval in the US and EU or after product development is discontinued. There are circumstances that may prevent MSD from sharing requested data, including country‐ or region‐specific regulations. If the request is declined, it will be communicated to the investigator. Access to genetic or exploratory biomarker data requires a detailed, hypothesis‐driven statistical analysis plan that is collaboratively developed by the requestor and MSD subject matter experts; after approval of the statistical analysis plan and execution of a data‐sharing agreement, MSD will either perform the proposed analyses and share the results with the requestor or will construct biomarker covariates and add them to a file with clinical data that are uploaded to an analysis portal so that the requestor can perform the proposed analyses.
