# Supplementary material for: Biomarkers predictive of response to pembrolizumab in head and neck cancer
Source: Cancer Med. 2022 Dec 7;12(6):6603–14. doi: 10.1002/cam4.5434 (PMC10067081; doi:10.1002/cam4.5434)

SUPPLEMENTARY MATERIAL

**SUPPLEMENTARY TABLE S1** Joint modeling of biomarkers

|  | **TMB and PD-L1 CPS** | | **TMB and T-cell–inflamed GEP** | |
| --- | --- | --- | --- | --- |
| Biomarker | TMB | CPS | TMB | T-cell–inflamed  GEP |
| One-sided *P* | 0.0007 | 0.0011 | 0.0002 | <0.0001 |

Abbreviations: CPS, combined positive score; GEP, gene expression profile; TMB, tumor mutational burden.

**SUPPLEMENTARY TABLE S2** HPV status by p16-IHC vs WES

|  | **HPV-negative**^b^  **p16-IHC, *n/N*** | **HPV-positive**  **p16-IHC, *n/N*** | **Total HPV WESc,**  ***n/N*** |
| --- | --- | --- | --- |
| HPV-negative WES | 164 | 13 | **177/256 (69%)** |
| HPV-positive WES | 35 | 44 | **79/256 (31%)** |
| Total HPV p16-IHC^a,b^ | 199/256 (78%) | 57/256 (22%) | — |

Abbreviations: HPV, human papillomavirus; IHC, immunohistochemistry; WES, whole exome sequencing.

^a^HPV status by p16-IHC was missing for one patient.

^b^Oropharynx includes some patients assumed to be HPV-negative because testing was limited to the oropharynx and non-oropharyngeal HNSCC tumors were considered HPV-negative.

^c^Defined as >20 reads mapping to HPV genome, all tumor sites.

**SUPPLEMENTARY TABLE S3** The interaction effect of each biomarker by HPV status for association with ORR by HPV assay

| **Biomarker** | **HPV Assay Used** | | | |
| --- | --- | --- | --- | --- |
|  | **WES** | | **p16** | |
|  | **Nominal *P*** | **Adjusted *P*** | **Nominal *P*** | **Adjusted *P*** |
| TMB | 0.7048 | 0.7048 | 0.4206 | 0.4206 |
| T-cell–inflamed GEP | 0.1118 | 0.2236 | 0.0259 | 0.0777 |
| PD-L1 CPS | 0.0150 | 0.0450 | 0.1295 | 0.2590 |

Note: Adjusted *P* values were calculated based on a Hochberg Step-up procedure to control the family-wise error rate for three tests within each HPV assay type.

Abbreviations: CPS, combined positive score; GEP, gene expression profile; HPV, human papillomavirus; TMB, tumor mutational burden.

**SUPPLEMENTARY FIGURE S1** Biomarker distribution by HPV status for (A) TMB, (B)

T-cell–inflamed GEP, and (C) PD-L1 CPS. CPS, combined positive score; GEP, gene expression profile; HPV, human papillomavirus; TMB, tumor mutational burden; WES, whole exome sequencing.


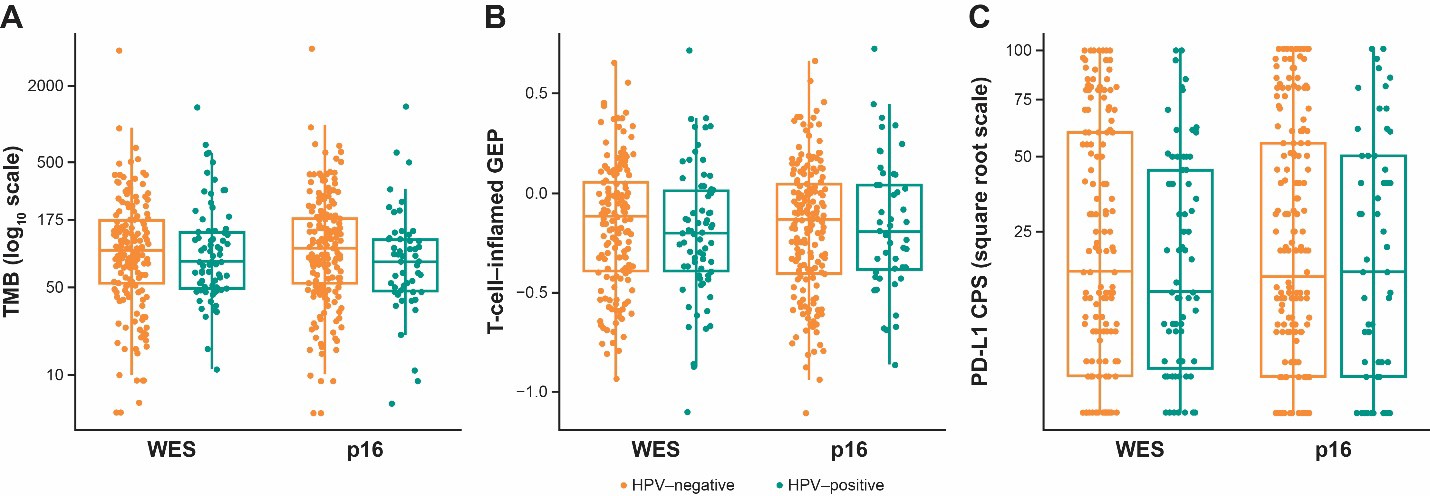


**SUPPLEMENTARY FIGURE S2** Association between biomarkers and response by HPV status for (A) TMB, (B) T-cell–inflamed GEP, and (C) PD-L1. CPS, combined positive score; CR, complete response; GEP, gene expression profile, HPV, human papillomavirus; NR, nonresponder; PD-L1, programmed death ligand 1; PR, partial response; R, responder; TMB, tumor mutational burden; WES, whole exome sequencing.


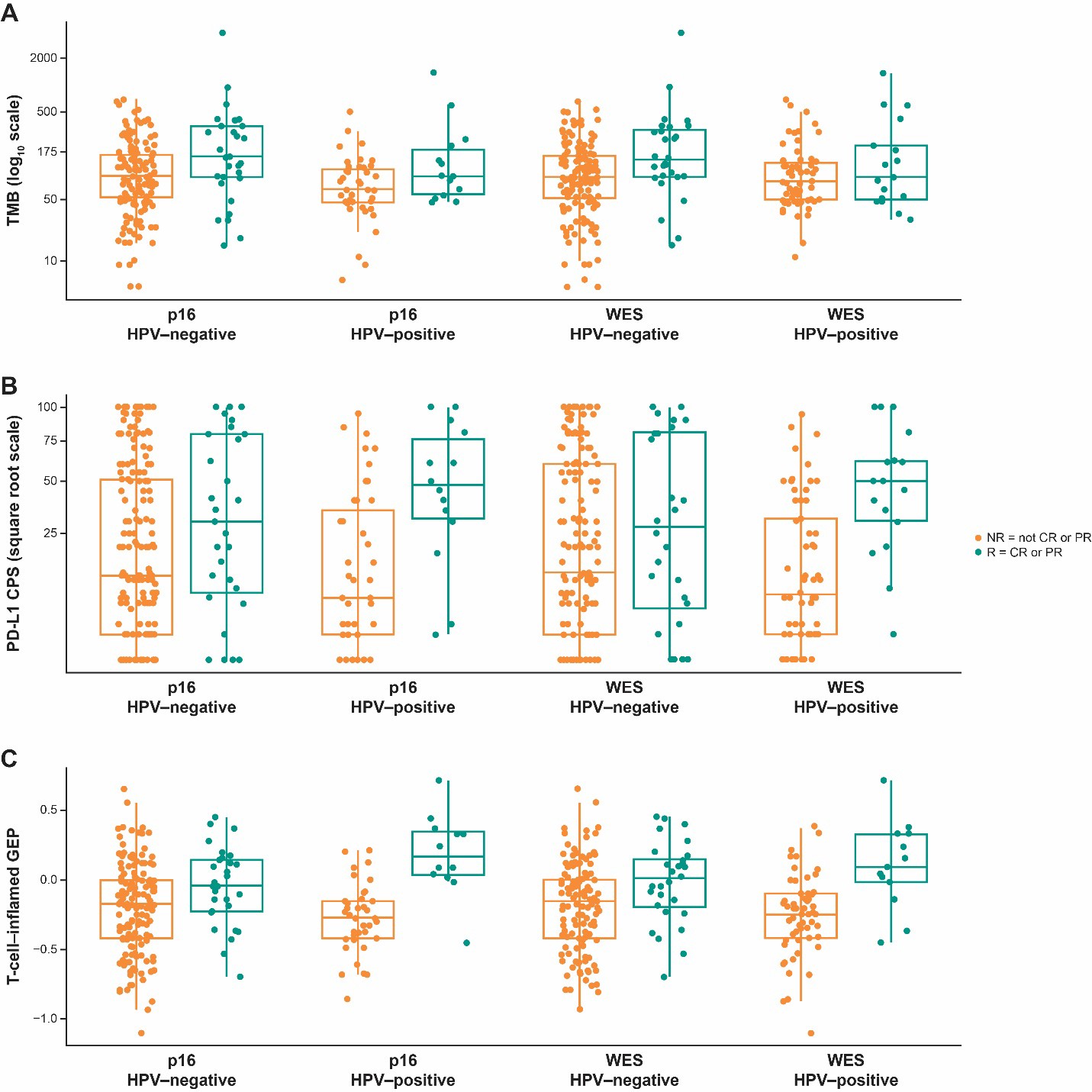

Supplement: Supplementary file 1 — Appendix S1 [file CAM4-12-6603-s001.docx]
